# Combining transformer and 3DCNN models to achieve co-design of structures and sequences of antibodies in a diffusional manner

**DOI:** 10.1016/j.jpha.2025.101267

**Published:** 2025-03-15

**Authors:** Yue Hu, Feng Tao, Jiajie Xu, Wen-Jun Lan, Jing Zhang, Wei Lan

**Affiliations:** a School of Bioengineering, Qilu University of Technology (Shandong Academy of Sciences), Jinan, 250300, China; b Kyiv College, Qilu University of Technology (Shandong Academy of Sciences), Jinan, 250300, China; c The Second Affiliated Hospital of Zhejiang University, School of Medicine, Zhejiang University, Hangzhou, 310053, China; d Binjiang Institute of Zhejiang University, Hangzhou, 310053, China; e College of Traditional Chinese Medicine, Xinjiang Medical University, Urumqi, 830011, China

## Abstract

• AlphaPanda: A novel AI algorithm for antibody design combining transformer, 3DCNN, and diffusion models.
• Captures global and local structural details for precise antibody-antigen interaction modeling.
• Enables flexible antibody design with potential for broader protein design applications.

## Graphical abstract

**Figure:**
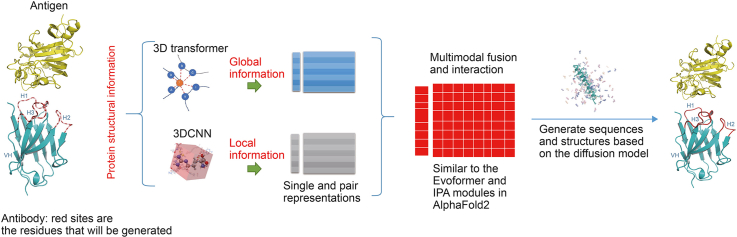

AlphaPanda (AlphaFold2 [1] inspired protein-specific antibody design in a diffusional manner) is an advanced algorithm for designing complementary determining regions (CDRs) of the antibody targeted the specific epitope, combining transformer [2] models, 3DCNN [3], and diffusion [4] generative models. Transformers capture global information and pairwise interactions, while 3DCNNs focus on local structural features and pairwise or non-pairwise interactions, requiring less data to training it. The diffusion model effectively generates sequences and structures, avoiding the limitations of autoregressive models or self-consistent iteration methods. In the generation mode of autoregression, the upstream errors tend to accumulate in the downstream errors. The generation method of self-consistent iteration does not have an effective function to guide the direction of generation. The program simultaneously generates alpha-carbon atoms of the main chain, main chain orientations, and residue sequence types, effectively achieving flexible main chain design, yielding excellent performance for the CDRs of antibodies design and broader protein design tasks. The source code is available on GitHub: https://github.com/YueHuLab/AlphaPanda.

Our model specifically focused on designing the CDRs, which were crucial for antibody affinity and specificity (Fig. 1). Unlike the constant (Fc) regions, which were structurally conserved and less relevant for antigen recognition, the CDRs played a key role in optimizing antibody performance. By concentrating on the CDR loops rather than the full antibody, we achieved a more focused sampling of the binding interface and reduced the complexity of designing the sequence and structure space. This approach aligned with traditional experimental methods and AI-driven strategies like diffab [5], which also prioritized CDR design for improved affinity. In our research, we utilized the AlphaPanda software for the design of the CDRs of antibodies, which demonstrated a relatively high degree of stability, as evidenced by the data summarized in Table 1, Supplementary text and Supplemental Files 1 and 2. In a case study involving the antibody with PDB ID: 7XJF, we successfully generated 500 different designs for the H_CDR3 region. Among these designs, only two exhibited notably high root mean square deviation (RMSD) values, which we identified as resulting from flawed design structures upon closer inspection. This suggested that the majority of the H_CDR3 designs were stable and well-formed, demonstrating the potential robustness of our methodology. AlphaPanda achieved lower RMSD values compared to diffab [5] (a transformer and diffusion based antibody design method) in this case, indicating a closer structural alignment with the original configuration.

**Fig. 1:**
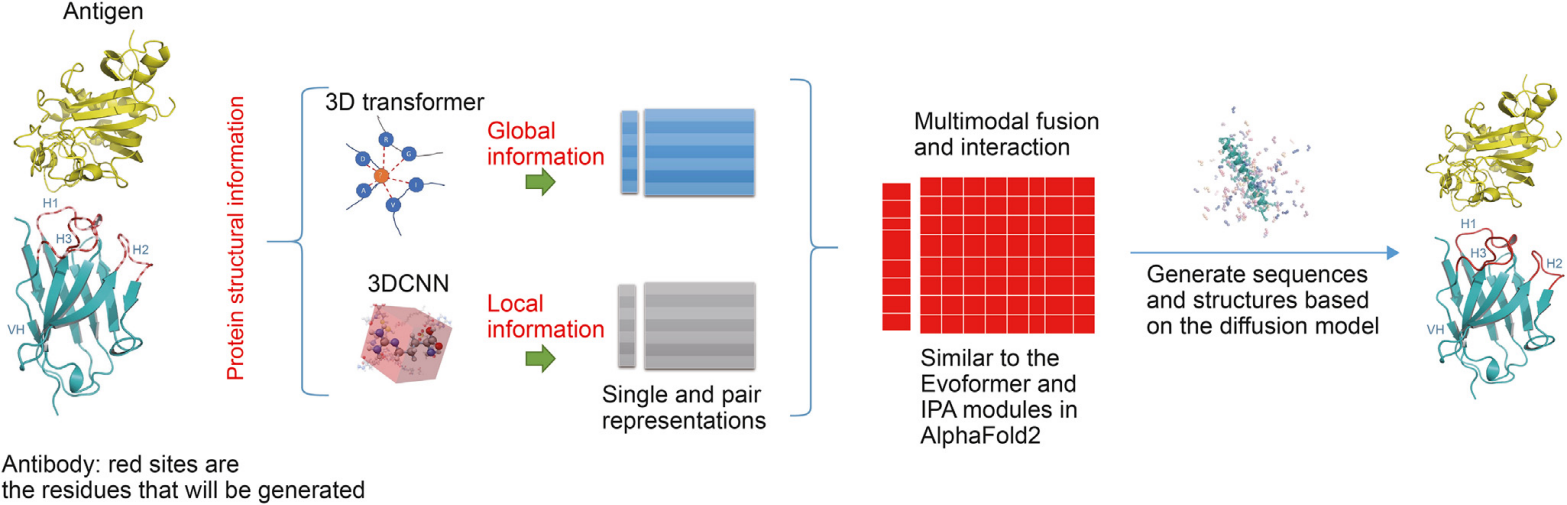
The AlphaPanda algorithm workflow: Given a protein target epitope, AlphaPanda aims to design the complementary determining regions (CDRs) of the antibody. Inspired by AlphaFold2, this algorithm combines transformer models to capture global structural information, 3DCNN models to focus on local features, and a diffusion model to generate both sequences and structures.

**Table 1:** 7XJF complementary determining region (CDR)-Wise comprehensive summary.

CDR	Method	Meets RMSD count	Meets seqid count	Meets ΔΔG count	Meets all criteria count	Total count	chi2 all criteria	*P*_value all criteria
H_CDR1	AlphaPanda	182	200	0	0	200	N/A	N/A
H_CDR1	diffab	100	100	5	5	100		
H_CDR2	AlphaPanda	200	96	24	13	200	28.1004	1.15 × 10^−7^
H_CDR2	diffab	100	53	53	30	100		
H_CDR3	AlphaPanda	0	53	0	0	498	N/A	N/A
H_CDR3	diffab	0	13	1	0	100		
L_CDR1	AlphaPanda	198	200	3	3	200	101.6856	6.51 × 10^−24^
L_CDR1	diffab	100	100	49	49	100		
L_CDR2	AlphaPanda	194	198	141	136	200	28.28947	1.04 × 10^−7^
L_CDR2	diffab	100	90	39	35	100		
L_CDR3	AlphaPanda	104	198	0	0	200	N/A	N/A
L_CDR3	diffab	100	100	4	4	100		

NA: not applicable; RMSD: root mean square deviation.

To further extend our analysis, we conducted a broader comparison across all six CDR regions of the 7XJF complex. Specifically, we evaluated 200 sequences for each of the two additional H_CDRs (excluding H_CDR3) as well as the three L_CDRs, all designed using AlphaPanda, and compared them to 100 sequences for each CDR generated by diffab. The findings revealed that, beyond H_CDR3, diffab tended to outperform AlphaPanda in terms of RMSD. This trend was also observed in other examples, such as complexes 7b3o, 8hpk, and 8a67, where diffab generally showed lower RMSD values compared to AlphaPanda (Table 1, Supplementary text, and Supplemental Files 1 and 2).

Then, we established specific criteria for defining a well-designed antibody, which included having an RMSD of less than 1.5 Å (the length of a typical carbon-carbon bond), a sequence identity (seqid) greater than 30%, and a negative ΔΔG value, indicating favorable energetic stability (Table 1 and Supplemental Files 1 and 2). Using these criteria, we conducted a chi-square test to compare the effectiveness of AlphaPanda and diffab across different CDR regions. The results indicated that for most CDRs, there was a higher proportion of well-designed antibodies generated by diffab compared to AlphaPanda. Specifically, diffab outperformed AlphaPanda in several regions, showing slightly better structural stability and sequence consistency. However, both methods faced challenges in the H_CDR3 region, with neither achieving a notably high success rate.

Interestingly, in the L_CDR2 region of the 7XJF (Table 1) and 8HPK complexes, AlphaPanda demonstrated a significantly higher proportion of well-designed antibodies, particularly regarding sequence identity (seqid) and stability (ΔΔG), highlighting a specific advantage for AlphaPanda in this region. For the L_CDR2 design, both methods performed well in the 7B3O complex, with diffab showing an edge in designing H_CDR2. Regarding the 8A67 complex, which is a nanobody, both methods generally showed average performance, although AlphaPanda had a slight advantage in H_CDR2.

AlphaPanda provides a significant advancement in the CDRs of antibodies design by integrating transformer models, 3DCNNs, and diffusion generative models. It consistently showed an advantage in generating diverse and stable antibodies, especially in flexible regions such as L_CDR2. While diffab demonstrated better performance in generating natural-like structures for certain regions, AlphaPanda excelled in achieving sequence diversity and favorable interaction profiles, making it highly suited for therapeutic applications that require novel binding solutions. We also conducted de novo testing on the AQP4 water channel, a key target involved in brain edema, focusing on its extracellular channel epitopes. The designed nanobodies were manually docked using PyMOL to evaluate spatial compatibility and validated through structural predictions on the AlphaFold3 web server, with results showing three of eight successful candidates targeting the desired epitopes (Supplementary text: 1.6 De novo design of nanobodies targeting AQP4 for brain edema). Despite the promising results, one major limitation of this study is the lack of experimental validation. While *in silico* performance was strong, experimental binding assays, protein stability tests, and immunogenicity analyses are essential to confirm the therapeutic viability of AlphaPanda's designed CDRs of antibodies. Future efforts will focus on collaborative experimental validation to further refine AlphaPanda and ensure its robustness for real-world antibody design. This study underscores AlphaPanda's strengths in combining deep learning approaches to tackle antibody design challenges, offering a versatile platform capable of innovating beyond current capabilities in therapeutic antibody development.

## CRediT authorship contribution statement

**Yue Hu:** Writing – original draft, Software, Methodology, Conceptualization. **Feng Tao:** Software, Methodology, Data curation, Conceptualization. **Jiajie Xu:** Validation, Software. **WenJun Lan:** Supervision, Methodology. **Jing Zhang:** Supervision, Methodology. **Wei Lan:** Writing – review & editing.

## Declaration of competing interest

The authors declare that there are no conflicts of interest.
