# Supplementary material for: Combining transformer and 3DCNN models to achieve co-design of structures and sequences of antibodies in a diffusional manner
Source: J Pharm Anal. 2025 Mar 15;15(6):101267. doi: 10.1016/j.jpha.2025.101267 (PMC12246596; doi:10.1016/j.jpha.2025.101267)
Supplement: Supplementary Text [file mmc3.docx]

**Supplementary Text for "Combining Transformer and 3DCNN Models to Achieve Co-Design of Structures and Sequences of Antibodies in a Diffusional Manner"**

**1. Supplementary Method**

**1.1 The keys of AlphaPanda**

The primary focus of our method is to combine a transformer and a 3DCNN to extract detailed information from the antibody-antigen complex environment and apply a diffusion model for generating sequences and structures. Specifically, our approach builds upon the diffab framework, integrating the protein design capabilities of the 3DCNN module and incorporating updates from AlphaFold2’s transformer module. Diffab, first presented at NeurIPS 2022, is a pioneering tool for generating antibodies targeting specific antigens, utilizing diffusion probabilistic models and equivariant neural networks for sequence-structure co-design, structure prediction, and optimization of complementarity-determining regions (CDRs). The 3DCNN model, on the other hand, uses deep learning to analyze protein structures. By combining diffab’s design capabilities with 3DCNN’s structural insights, we aim to create a more comprehensive platform for antibody design, protein structure analysis, and broader protein engineering tasks, thereby expanding the scope and accuracy of protein design research.

A key aspect of our approach is the seamless integration of the 3DCNN, particularly in establishing a consistent reference frame or basis for structural analysis. This involves constructing a coordinate system based on atomic positions, ensuring compatibility with the coordinate system used by the transformer module. The orientations of the amino acid residues differed between the two software tools. We have unified the residue representations to match the orientations used in the diffab program. Additionally, our design includes an interface for protein language models, adding flexibility to the overall protein design process in the future.

The software is open source and licensed by Apache 2.0. Everyone can get the source code from github (https://github.com/YueHuLab/AlphaPanda).We believe that making the code publicly available will contribute to advancing the entire research field. Our integration and improvements of the code are reflected in the following tree, which outlines the structure of our approach. Additionally, we have provided an interface for the future integration of protein language models, offering the potential for further enhancement and flexibility in protein design tasks (https://github.com/YueHuLab/AlphaPanda/tree/AlphaPanda_PLM).

**1.2 Details of AlpahPanda**

The following is a brief introduction to the main ideas and algorithms of AlphaPanda (**Algorithm 1**). Proteins are made up of amino acid residues. An amino acid residue can be represented by its sequence type$s_{i}\in\left\{ \mathrm{ACDEFGHIKLMNPQRSTVWY} \right\}$, its atomic coordinates of alpha-carbon $C_{\alpha}\boldsymbol{x}_{i}\in\mathbb{R}^{3}$ and its frame orientation$\boldsymbol{O}_{i}\in SO\left（ 3 \right）$ respectively. The latter two items are structural information. So we represent both sequence and structural information simultaneously in this way. In (nano) antibody design, targeting antigen epitopes requires determining the general position of antigen-antibody complex binding. The primary task is to generate the sequence and structure of the antibody's CDR region, given the rest of the antigen-antibody complex. We treat both the given protein information and the sampled information during generation as context features. Environmental information is embedded using transformer and 3DCNN models. The transformer captures residue-level and interaction-level details (residue type, secondary structure, atomic coordinates, spatial interactions), while the 3DCNN focuses solely on the spatial positions of surrounding atoms, ignoring protein chain distinctions. This approach potentially simplifies the interaction design, particularly for long-chain or multi-chain proteins.

Dependent on environmental information, the structure and sequence of CDR regions $\mathcal{R=}\left\{ (s_{j},\boldsymbol{x}_{j},\boldsymbol{O}_{j}) \right\}_{j=l+1}^{l+m}$are generated by generation diffusion iteration. Together with known ones, the structural information of the remaining protein antigen and antibody complexes$\mathcal{C=}\{(s_{i},\boldsymbol{x}_{i},\boldsymbol{O}_{i})|i\in\left\{ 1 . . . N \right\}\backslash\{l+1, . . . , l+m\}\}$ , constitutes the amino acid information of the entire protein$\mathcal{P\leftarrow}\left\{ \left( s_{i},\boldsymbol{x}_{i},\boldsymbol{O}_{i} \right) \right\}_{i=1}^{N}$.

The generated information of these amino acids of proteins $\mathcal{P\leftarrow}\left\{ \left( s_{i},\boldsymbol{x}_{i},\boldsymbol{O}_{i} \right) \right\}_{i=1}^{N}$ is transmitted through a IPA like module of Alphafold2 into the Transformer model. Meanwhile, the generated information$\mathcal{P\leftarrow}\left\{ \left( s_{i},\boldsymbol{x}_{i},\boldsymbol{O}_{i} \right) \right\}_{i=1}^{N}$ is also transmitted to the 3DCNN model by updating the protein structure and sequence.

**The overall flow is shown in Algorithm 1.**

**Algorithm 1**

| **AlphaPanda model inference** | | | | |  |
| --- | --- | --- | --- | --- | --- |
| def Inference ($\left\{ \boldsymbol{m}_{i} \right\}, \left\{ \boldsymbol{z}_{ij} \right\},\mathcal{C=}\{(s_{i},\boldsymbol{x}_{i},\boldsymbol{O}_{i})\vert i\in\{1 . . . N\}\backslash\{l+1, . . . , l+m\}\}$, T) | | | | |  |
| # Enter annotation: single residue information $\left\{ \boldsymbol{m}_{i} \right\}\in\mathbb{R}^{N\times c_{m}}$and pair residues information.$\left\{ \boldsymbol{z}_{ij} \right\}\in\mathbb{R}^{N\times N\times c_{z}}$  # Enter annotation: complementary sequences and structure information $\mathcal{C}$ (antigen-antibody complex, excluding CDR region)  # Enter annotation: Sequence type $s_{i}\in\left\{ \mathrm{ACDEFGHIKLMNPQRSTVWY} \right\}$  # Enter annotation: Carbon atomic coordinates $C_{\alpha}\boldsymbol{x}_{i}\in\mathbb{R}^{3}$  # Enter annotation: Residue orientation $\boldsymbol{O}_{i}\in SO\left（ 3 \right）$  # Enter annotation: back propagation steps T | | | | |  |
| 1: $\boldsymbol{m}_{i}^{T},\boldsymbol{z}_{ij}^{T}$←Linear$\left（ \boldsymbol{m}_{i} \right）$, Linear$\left（ \boldsymbol{z}_{ij} \right）$ | | | | $\#\boldsymbol{m}_{i}^{T}\in\mathbb{R}^{c},z_{ij}^{T}\in\mathbb{R}^{c}$ |  |
| 2:$\mathcal{R}^{T}\leftarrow\left\{ \left( \boldsymbol{s}_{j}^{T},\boldsymbol{x}_{j}^{T},\boldsymbol{O}_{j}^{T} \right)_{j=l+1}^{l+m} \right\}\leftarrow\left\{ \left( \frac{1}{20}\cdot\mathbf{1},\left( 0,0,0 \right),\boldsymbol{I}_{3} \right) \right\}_{j=l+1}^{l+m}$ | | | | Initialize CDR region sequence and structure information$\mathcal{R}^{T}$ |  |
| # tag i represents the residue number of the entire protein $\mathcal{P}$ and tag j represents the residue number of the CDR region $\mathcal{R}$ | | | | |  |
| 3：由$\mathcal{P}^{T}=\mathcal{R}^{T}+\mathcal{C}$，得$\mathcal{P}^{T}\leftarrow\left\{ \left( \boldsymbol{s}_{i}^{T},\boldsymbol{x}_{i}^{T},\boldsymbol{O}_{i}^{T} \right)_{i=1}^{N} \right\}\leftarrow\left\{ \mathcal{R}^{T}+\mathcal{C} \right\}$ | | | | # Initialize the entire protein sequence and structure information$\mathcal{P}^{T}$ |  |
| **# Begins the generation diffusion process** | | | |  |  |
| 4: for all t ∈ [T, . . . , 0] do # Diffusion model t=T represents a priori state, t=0 represents a true generated state | | | | |  |
| 5: $\boldsymbol{m}_{i}^{t},\boldsymbol{z}_{ij}^{t}$←MultiModel$\left（ \boldsymbol{m}_{i}^{t},\mathrm{transformer}\left( \mathcal{P}^{T} \right)，3DCNN\left( \mathcal{P}^{T} \right) \right）$, $\mathrm{MultiModel}\left（ \boldsymbol{z}_{ij}^{t},transformer\left( \mathcal{P}^{T} \right)，3DCNN\left( \mathcal{P}^{T} \right) \right）$ #combined transformer and 3DCNN | | | | |  |
| **# Evoformer Module Begins: Interact with information**$\left\{ \boldsymbol{m}_{i}^{t} \right\}\left\{ \boldsymbol{z}_{ij}^{t} \right\}$ | | | | |  |
| 6： **for all h ∈ [0, . . . , H-1] do# H layers** | | | | |  |
| 7： $\boldsymbol{m}_{i}^{t}$←MHA$\left( \left\{ \boldsymbol{m}_{i}^{t} \right\},\left\{ \boldsymbol{z}_{ij}^{t} \right\} \right)$ # Multi-Head Self-Attention Mechanism | | | | |  |
| 8: $\boldsymbol{z}_{ij}^{t}\leftarrow\boldsymbol{z}_{ij}^{t}+\mathrm{Linear}\left（ \boldsymbol{m}_{i}^{t}\bigotimes\boldsymbol{m}_{j}^{t} \right）$ | | |  | |  |
| # In the next two steps, optimize the structure by using triangular geometric constraints | | |  | |  |
| 9:  $\boldsymbol{z}_{ij}^{t}\leftarrow\boldsymbol{z}_{ij}^{t}+TriangleMultiplication\left( \left\{ \boldsymbol{z}_{ij}^{t} \right\} \right)$ **#AlphaFold2** | | | | |  |
| 10: $\boldsymbol{z}_{ij}^{t}\leftarrow\boldsymbol{z}_{ij}^{t}+TriangleAttention\left( \left\{ \boldsymbol{z}_{ij}^{t} \right\} \right)$ **#AlphaFold2** | | | | |  |
| 11: End for | | |  | |  |
| **# End of Evoformer module** | | |  | |  |
| # Invariant point attention (IPA) module, interactive information among $s_{i},\boldsymbol{x}_{i},\boldsymbol{O}_{i},\boldsymbol{m}_{i},\boldsymbol{z}_{ij}$  # IPA Module Start | | | | |  |
| 12:$\boldsymbol{s}_{i}^{t-0.5}\leftarrow\mathrm{MLP}_{e}\left（ \boldsymbol{s}_{i}^{t} \right）$ | | | # Initialize $\boldsymbol{s}_{i}^{t-0.5}\in\mathbb{R}^{c}$ | |  |
| 13： **for all k ∈ [0, . . . , K-1] do** | | | # Shared K Layers | |  |
| 14： $\boldsymbol{m}_{i}^{t},\boldsymbol{z}_{ij}^{t}\leftarrow IPA\left( \left\{ \boldsymbol{m}_{i}^{t} \right\},\left\{ \boldsymbol{z}_{ij}^{t} \right\},\left\{ \boldsymbol{s}_{i}^{t-0.5} \right\},\left\{ \boldsymbol{x}_{i}^{t} \right\},\left\{ \boldsymbol{O}_{i}^{t} \right\} \right)$ **# IPA** module in AlphaFold2 | | | | | |
| 15: End for | | | | |  |
| # IPA module end | | | | |  |
| 16:$\boldsymbol{m}_{i}^{t-1},\boldsymbol{z}_{ij}^{t-1}\leftarrow\boldsymbol{m}_{i}^{t},\boldsymbol{z}_{ij}^{t}$ | | | | |  |
| # Generate diffusion module: update the sequence and structure of CDR region in the way of diffusion generation, which is recorded as $\mathcal{R}^{t}\leftarrow\left\{ \left( \boldsymbol{s}_{j}^{t},\boldsymbol{x}_{j}^{t},\boldsymbol{O}_{j}^{t} \right)_{j=l+1}^{l+m} \right\}$  # The CDR region is tagged j and the whole protein is tagged i | | | | |  |
| 17:${\hat{\boldsymbol{x}}}_{j}^{t}\leftarrow\mathrm{MLP}_{x}\left( \boldsymbol{m}_{i}^{t-1},\boldsymbol{m}_{i}^{T} \right)$ | # Updated location in local coordinates $C_{\alpha}$ | | | |  |
| 18: $\boldsymbol{x}_{j}^{t-1}\leftarrow\boldsymbol{x}_{j}^{t}+\boldsymbol{O}_{i}^{t}{\hat{\boldsymbol{x}}}_{j}^{t}\# Updated location in local coordinates C_{\alpha}$ | | | | |  |
| 19:${\hat{\boldsymbol{O}}}_{j}^{t}\leftarrow convert\left( \mathrm{MLP}_{o}\left( \boldsymbol{m}_{i}^{t-1},\boldsymbol{m}_{i}^{T} \right) \right)$ | | # Update residues orientations | | |  |
| 20:$\boldsymbol{O}_{j}^{t-1}\leftarrow\boldsymbol{O}_{j}^{t}{\hat{\boldsymbol{O}}}_{j}^{t}$ | | | # Update residues orientations | |  |
| 21:$\boldsymbol{s}_{j}^{t-1}\leftarrow s\mathrm{oftmax}\left( \lambda\cdot\mathrm{MLP}_{s}\left( \boldsymbol{m}_{i}^{t-1},\boldsymbol{m}_{i}^{T},\boldsymbol{s}_{i}^{t-0.5} \right) \right)$ | | | # Update sequence | |  |
| 22:$\mathcal{R}^{t-1}\leftarrow\left\{ \left( \boldsymbol{s}_{j}^{t-1},\boldsymbol{x}_{j}^{t-1},\boldsymbol{O}_{j}^{t-1} \right)_{j=l+1}^{l+m} \right\}$ | | | # Update CDR region sequence and structure | |  |
| 23:$\mathcal{P}^{t-1}\leftarrow\left\{ \left( \boldsymbol{s}_{i}^{t-1},\boldsymbol{x}_{i}^{t-1},\boldsymbol{O}_{i}^{t-1} \right) \right\}_{i=1}^{N}\leftarrow\left\{ \mathcal{R}^{t-1}+\mathcal{C} \right\}$ | | | # Update the sequence and structure of the whole protein | |  |
| 24.: End for | | |  | |  |
| **# End of generation diffusion process** | | |  | |  |
| 25: Return: $\mathcal{P}^{0}$and$\mathcal{R}^{0}$ | | | | |  |

**1.3 Training details**

The diffusion probabilistic model is a generative model involving two Markov processes: a forward process that gradually adds noise, transforming the real data distribution into a prior (typically Gaussian), and a reverse process that denoises the prior to reconstruct the original distribution. By treating generation as a stepwise denoising process, training this reverse process allows for effective protein sequence-structure co-generation. This involves using the forward process to compute the posterior distribution and optimizing the model by minimizing the difference between the real and generated distributions. We denote the sequence-structure state of the entire CDR region by representing the state of amino acid residue j at step T. Step t=0 represents the true state (natural CDR region used for training), and t=T represents the prior state distribution (generally Gaussian). The forward process progresses from t=0 to t=T, while the reverse generation moves from t=T to t=0. In the training process, we first select a time step t, calculate the posterior probability q, estimate the posterior p using an MLP network, and compute the loss between them. This loss is back propagated to update the model parameters, including fine-tuning the protein sequence language model, until convergence. Training is performed sequentially: starting with a protein-protein interaction complex database, followed by an antigen-antibody structure database, and finally fine-tuning with a nanobody-antigen structure database. Our optimization objectives are represented by Equations (1), (2), (3), and (4).

A. Diffusion Model of Amino Acid Types

$L_{\mathrm{type}}^{t}\mathbb{=E}_{\mathcal{R}^{t}\sim p}\left[ \frac{1}{m}\sum_{j} D_{\mathrm{KL}}\left( q\left. \left( \left. s_{j}^{t-1} \right|s_{j}^{t},s_{j}^{0} \right) \right\|{Conv1d}_{\mathrm{type}}\left( Transformer,3DCNN \right) \right) \right]$ (1)

B. Diffusion model of atomic coordinates$C_{\alpha}\boldsymbol{x}_{i}$

$L_{\mathrm{pos}}^{t}\mathbb{=E}\left[ \frac{1}{m}\sum_{j} \left\| \epsilon_{j}-{Conv1d}_{pos}\left( Transformer,3DCNN \right) \right\|^{2} \right]$ (2)

C. Frame Orientation Diffusion Process of Amino Acid$\boldsymbol{O}_{i}\in SO\left（ 3 \right）$

$L_{\mathrm{ori}}^{t}\mathbb{=E}\left[ \frac{1}{m}\sum_{j} \left\| \left（ \mathbf{O}_{j}^{0} \right）^{\top}{Conv1d}_{ori}\left( Transformer,3DCNN \right)\left[ j \right]-\boldsymbol{I} \right\|_{F}^{2} \right]$ (3)

D. Training process

$L=\mathbb{E}_{t\sim Uniform\left( 1\ldots T \right)}\left[ {\alpha L}_{\mathrm{type}}^{t}+{\beta L}_{\mathrm{pos}}^{t}+\gamma L_{\mathrm{ori}}^{t} \right]$ (4)

**1.3.1 Training Data and Model Configuration**

For our training process, we utilized the SAbDab (Structural Antibody Database) to gather high-quality structural data of antibodies and antibody-antigen complexes. This database is a rich resource that includes 13,073 antibody-antigen complex entries, which were instrumental in developing and validating our computational models. The structural data (approximately 4.6 GB) and a summary of all available entries can be directly accessed from the SAbDab website. To ensure reproducibility, we specify that the version used for this study was downloaded on March 20, 2023, and it is available for reference at the following link: https://huggingface.co/datasets/YueHuLab/AlphaPanda_training_dataset.

The dataset was divided into a training set and a validation set to facilitate effective model evaluation. Initially, we shuffled the combined dataset using a fixed seed (2023) to ensure reproducibility. After shuffling, the first 20 samples were assigned to the validation set, with the remaining samples used for training. This division allows for continuous evaluation of the model's performance on unseen data, helping to adjust training and prevent overfitting. The training data was used for parameter updates, while the validation data provided periodic checkpoints for performance monitoring.

The test data was released later, on May 21, 2023. This time separation ensures that there is no overlap between the training and test datasets. We have taken careful measures to maintain this separation, avoiding any potential data leakage and preserving the integrity of the model’s evaluation process.

**1.3.2 Model Training Configuration**

Our training setup was designed for balanced and effective optimization. The model architecture features a resolution feature dimension of 128 and a pairwise feature dimension of 64, with a diffusion process encompassing 100 steps and a network configuration of 6 layers, emphasizing both structural and sequence training. A learning rate of 0.001 was used, with the Adam optimizer configured with β1=0.9, β2=0.999, and no weight decay to support steady learning dynamics. To ensure training stability, a maximum gradient norm of 100.0 was applied, which helped prevent excessively large gradient updates.

The training process was conducted over a maximum of 600,000 iterations, with validation checks every 1,000 iterations to regularly assess model performance. The loss function is a weighted combination of three components—rotation, position, and sequence—each weighted equally at 1.0, ensuring that all aspects of prediction are optimized evenly. To adjust the learning rate dynamically, a plateau scheduler was used, reducing the learning rate by a factor of 0.8 if no improvement was seen over 10 validation cycles, with a minimum learning rate of 5e-6. This comprehensive configuration ensures that the model converges effectively while maintaining robustness and generalization capabilities.

**1.4 Evaluation Metrics**

The number of residues in the generated sequences and structures may differ from those in the natural antibody, especially when comparing similarity metrics such as RMSD (for structural comparison), AAR (for sequence comparison), and binding free energy (ΔG) for evaluating interaction strength. It is crucial to clarify these methods to ensure a proper understanding of how these comparisons are performed.

**1.4.1 RMSD (Root Mean Square Deviation)**

RMSD is used to assess the structural similarity between the generated structure and a reference structure, focusing on the alignment of Cα atoms. The RMSD calculation employs a dynamic programming approach to align residues, particularly useful when the lengths of the generated and reference structures differ.

RMSD Calculation

1. Input Parameters: Two lists of residues representing the Cα atoms of the reference structure and the generated structure.

2. Determining Shorter and Longer Residue Lists: To optimize the computation, the shorter of the two lists is identified.

3. Distance Calculation: A helper function d(i, j) calculates the squared distance between the Cα atoms of residues at index i in the shorter list and index j in the longer list:

$d\left( i, j \right)= \Sigma\left( \mathrm{coord}_{i}- \mathrm{coord}_{j} \right)^{2}$

Here,$\mathrm{coord}_{i}$ and $\mathrm{coord}_{j}$ represent the coordinates of the Cα atoms in the respective structures.

4. Dynamic Programming Table Initialization: A similarity distance matrix SD is initialized to store the squared distances, with initial values set to infinity.

5. Dynamic Programming Approach: The algorithm populates the SD matrix by iteratively calculating the minimal sum of distances:

$SD[i, j] = min(d(i, j) + SD[i+1, j+1], SD[i, j+1])$

This formula finds the optimal alignment by either aligning the next residues or skipping a residue in the longer list to minimize the total distance.

6. Final RMSD Calculation: The optimal alignment is derived from the minimum value in the SD matrix. The RMSD is then computed as:

$RMSD = \sqrt{\frac{min(SD)}{N}}$

**1.4.2 AAR (Amino Acid Recovery)**

AAR measures the sequence similarity between the generated and reference antibody CDR sequences. This metric is crucial in antibody design as it reflects how well the generative model retains the sequence features that are important for binding affinity.

AAR Calculation

1. Comparison of Sequences: The amino acid sequences of the generated CDR and the reference CDR are compared on a position-by-position basis.

2. Matching Amino Acids: For each position i, the amino acid in the generated sequence is compared to the corresponding amino acid in the reference sequence. If they match, that position is counted as a match.

3. AAR Formula:

$$AAR=\frac{Number of matching amino acids}{Total number of amino acids}*100\%$$

**1.4.3 Binding Free Energy (Δ**Δ**G)**

In evaluating the antibodies’ binding affinity, the present study focuses on designing the complementarity-determining regions (CDRs) for a given protein target epitope, where both the antigen and antibody binding sites are already defined. As a result, a full-scale docking procedure is not necessary. Instead, this work employs ΔΔG (change in Gibbs free energy) calculations, a well-established method for quantifying the thermodynamic stability—and therefore the binding affinity—of antibody-antigen complexes. Specifically, the Rosetta software suite developed by David Baker’s group is utilized to compute ΔΔG values. By concentrating on how alterations in the CDR regions affect the complex’s thermodynamic stability, binding affinity can be effectively assessed without performing a separate docking step. ΔΔG is calculated to evaluate the interaction strength between the antibody and the antigen, using PyRosetta’s InterfaceAnalyzerMover.

Binding Free Energy (ΔΔG) Calculation

1. Loading and Preparing the Structure: The antibody-antigen complex is loaded from a PDB file, and the chains are virtually separated.

2. Repacking Side Chains: The tool repacks the side chains at the interface to account for potential structural adjustments upon separation.

3. Computing the Binding Free Energy: The difference in free energy between the bound and unbound states (ΔG_separated) is computed:

$\Delta\Delta G = \Delta G\_complex - (\Delta G\_antibody + \Delta G\_antigen)$

The calculations for RMSD and AAR are implemented in the similarity.py file, while the energy calculations are in the energy.py file, both located in our repository at <https://github.com/YueHuLab/AlphaPanda/tree/main/AlphaPanda/tools/eval>. These scripts ensure accurate evaluation of structural similarity, sequence recovery, and interaction strength, providing a comprehensive analysis of the designed antibody models.

**1.5 Evaluated the results of the design**

We evaluated our methodology by designing complementary-determining region (CDR) sequences for several antibody-antigen complexes that were not part of our training or test database. Specifically, we used the crystal structure of the antibody-antigen protein complex with PDB ID 7xjf, released on May 31, 2023, as our primary test case. Initially, we generated 500 H_CDR3 sequences using the AlphaPanda software, allowing us to assess the performance and stability of the designs. In addition, as a control, we created 100 H_CDR3 sequences using diffab. For 7xjf, in addition to the initial 500 H_CDR3 sequences designed, we expanded the study to include 200 sequences each for two additional H_CDRs (excluding H_CDR3) and three L_CDRs using AlphaPanda. For comparison, we designed 100 sequences each for the same CDRs using diffab. These newly generated sequences allowed for a comprehensive analysis of the performance and stability of both AlphaPanda and diffab across different CDR regions.

To further expand our evaluation, we included three more complexes: 7b3o, 8hpk, and 8a67. For the 7b3o and 8hpk complexes, which consist of three H_CDRs and three L_CDRs, we designed 200 sequences for each CDR using AlphaPanda and 100 sequences for each CDR using diffab. The complex 8a67, which represents a nanobody structure containing only three H_CDRs, underwent the same evaluation process, with 200 sequences designed per CDR using AlphaPanda and 100 sequences designed per CDR using diffab.

**1.6 De Novo Design of Nanobodies Targeting AQP4 for Brain Edema**

The AQP4 (Aquaporin-4) water channel is a critical therapeutic target for brain edema, playing a key role in regulating water homeostasis within the central nervous system. Despite its importance, no nanobodies targeting AQP4 have been reported so far. To address this, we used computational approaches to design nanobodies targeting AQP4. Using chain A of the structure with PDB ID 3GD8 as the AQP4 template and the humanized nanobody NbBCII10 (FGLA mutant) framework (PDB ID 3EAK) as a starting point, we designed complementary-determining regions (CDRs) targeting key extracellular epitopes of AQP4, primarily the 141–151 region. The epitope was expanded to include 140–155, 57–69, 203–208, and 222–232, and the CDRs (Chothia numbering: H1, 26–32; H2, 52–56; H3, 95–102) were docked near these regions using PyMOL (manual dragging) (**Supplementary Figure 1**). After validating docking results and avoiding spatial clashes, we generated eight redesigned CDRs, three of which successfully bound near the epitope as validated using the AlphaFold3 (AF3) web server (**Supplementary Figure 2 and Supplementary Figure 3**). These promising results provide a foundation for developing nanobody-based therapies targeting AQP4 for brain edema treatment. The original results predicted by AlphaFold3 have also been uploaded as attachments, and they can be easily verified using the AlphaFold3 web server.


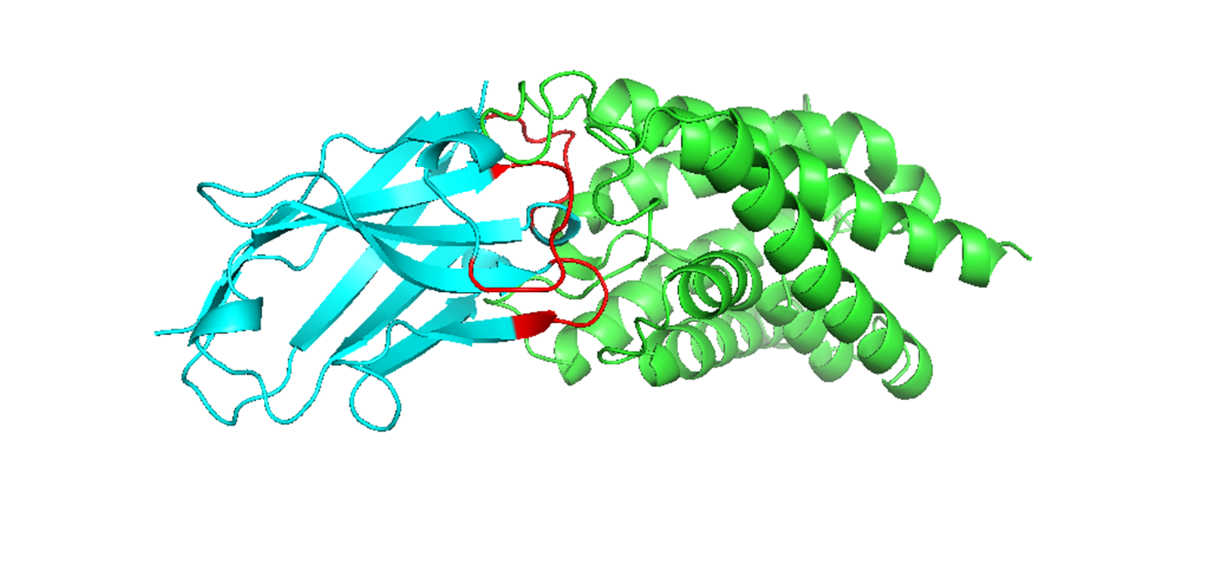


**Supplementary Figure 1: The initial manual docking results, the nanobody is shown in blue, the CDR regions are highlighted in red, and AQP4 is represented in green.**


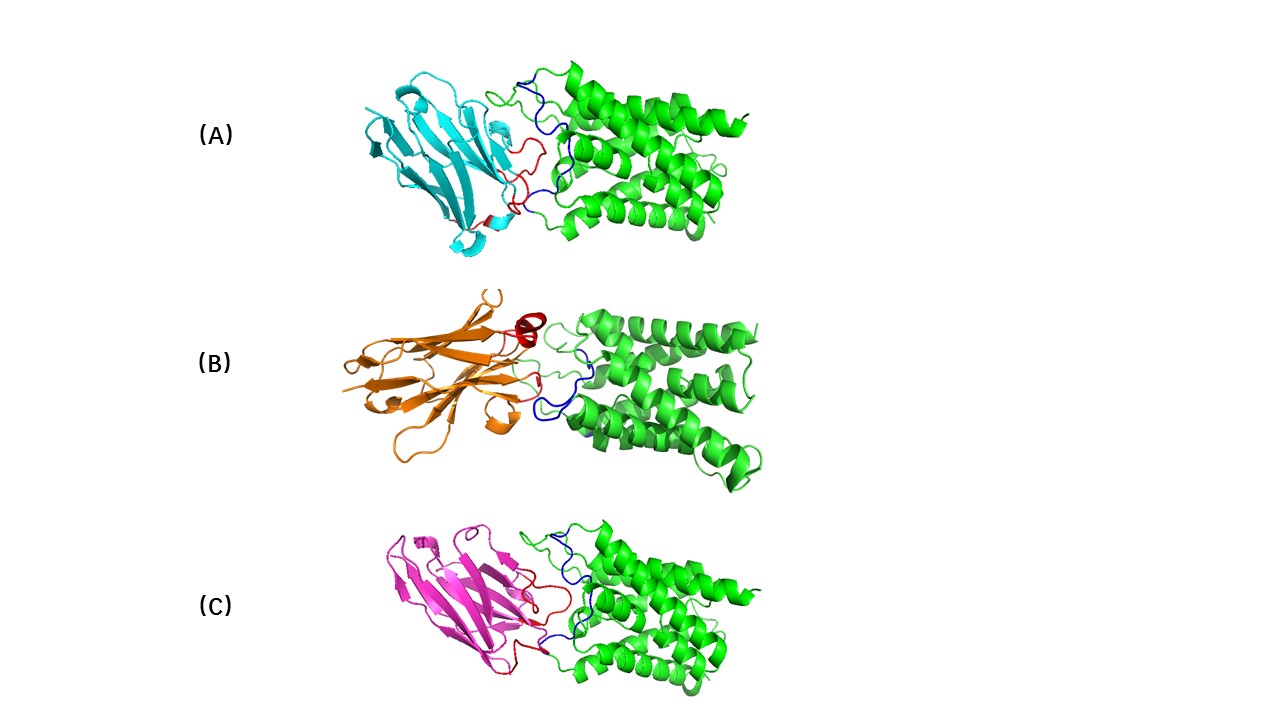


**Supplementary Figure 2: Using the AlphaFold3 webserver, the designed nanobody-antigen complexes were predicted. In panels A, B, and C, the orange, cyan, and magenta colors represent the designed nanobodies, while green represents the antigen AQP4. The red regions indicate the CDRs, and blue highlights the epitope region.**


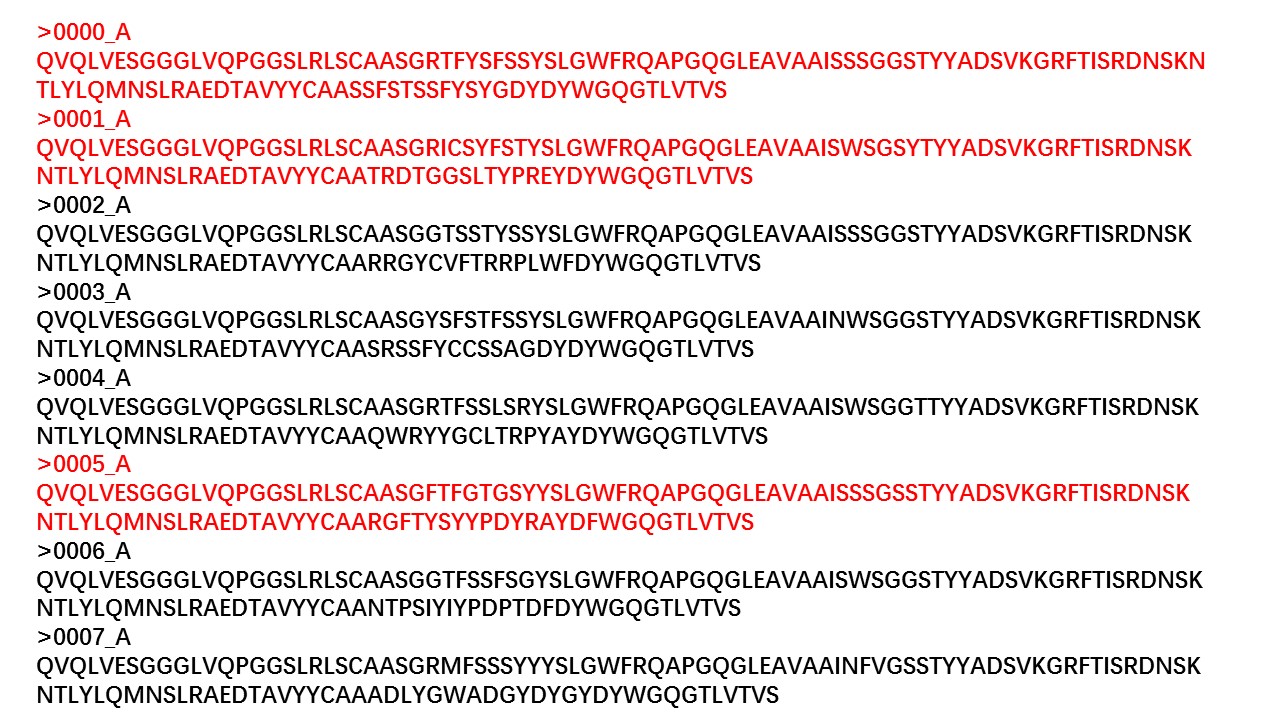


**Supplementary Figure 3: The eight designed sequences were predicted using the AlphaFold3 webserver, with those targeting the epitope region highlighted in red.**

**2. Supplementary Discussion: Comparative Insights, Limitations, and Future Perspectives**

Our findings reveal key insights into the comparative performance of AlphaPanda and diffab, highlighting their respective strengths and weaknesses. Firstly, AlphaPanda demonstrated the capability to design stable and well-structured antibodies, showcasing its ability to generate structurally reliable candidates. Secondly, statistical analysis using t-tests revealed that diffab consistently outperformed AlphaPanda in terms of RMSD, indicating superior stability in its designs. We further evaluated the success rates of both models based on a rigorous set of criteria, which highlighted specific contexts in which AlphaPanda exhibited clear advantages. Finally, while AlphaPanda's designs were characterized by greater structural diversity, diffab's generated antibodies showed closer resemblance to natural structures, underscoring a balance between innovation and biological relevance.

**2.1 Comparison and Limitations**

AlphaPanda represents a significant advancement in antibody and protein design by merging transformer models, 3D convolutional neural networks (3DCNN), and diffusion generative models. Compared to tools like AlphaFold 2 and 3, which excel at predicting protein structures, AlphaPanda has a different focus: designing antibody sequences and structures based on specific antigen structures and functions. While AlphaFold primarily solves the sequence-to-structure problem (protein folding), our approach tackles the inverse problem—generating sequences for a given structure.

Our method draws inspiration from AlphaFold 2, incorporating the transformer module to encode sequence information and capture the global context of antigen-antibody complexes. However, transformers have inherent limitations as they focus mainly on pairwise interactions and struggle to capture structural aspects such as chirality. To overcome these limitations, AlphaPanda integrates a 3DCNN module, which effectively captures local structural details and non-pairwise features, allowing for a more nuanced understanding of antigen-antibody interactions.

For sequence and structure generation, we employ a diffusion-based generative model. Unlike autoregressive models, which accumulate errors due to their sequential nature, or self-consistent iterative models, which often face issues with convergence and overfitting, the diffusion model provides an efficient and parallelizable approach. This makes it well-suited for the robust and complex requirements of antibody design.

However, one significant limitation of this study is the lack of experimental validation. Although AlphaPanda has demonstrated promising performance in designing antibodies with strong binding affinities in silico, it is essential to validate these designs in biological systems. Wet lab experiments that assess binding affinity, specificity, protein stability, immunogenicity, and potential off-target effects are crucial to confirming that the computationally generated antibodies function as expected in therapeutic settings. Future efforts will focus on collaborative experimental validation, refining the algorithm and ensuring its reliability for practical antibody design. Additionally, we observed that both diffab and AlphaPanda performed poorly on the H_CDR3 loop. This is likely due to the loop's considerable length and high flexibility, highlighting the need to incorporate more advanced computational methods in the future to improve success rates.

**2.2 Protein Language Model: Benefits and Limitations**

Many antibody design programs utilize protein or antibody language models to constrain the sampling space by fine-tuning on known antibody sequences. These models harness coevolutionary information, which has proven useful for general protein-protein interaction tasks. However, in the context of antibodies and antigens—such as those found in host-pathogen interactions—the concept of coevolution may not fully apply. Instead, antigen and antibody interactions involve a form of "negative coevolution," where the pathogen evolves to escape the antibody response. Thus, the conventional protein language model might provide only partial insights, and a significant portion of the critical information might remain inaccessible.

Nonetheless, it is important to acknowledge that protein language models can still offer valuable insights, as demonstrated by successes in unrelated proteins like orphan proteins. This suggests that, despite the limitations, protein language models could contribute useful general features and patterns to antibody design, albeit with reduced specificity compared to more targeted approaches. To facilitate this, we have also provided an interface for integrating the protein language model, enabling its seamless incorporation into the antibody design pipeline.

**2.3 The important of the frame orientation**

In our model, like diffab, we have incorporated an additional parameter—frame orientation—which plays a crucial role in both encoding side-chain information and capturing inter-residue interactions (**Supplementary Figure 4**). Unlike traditional methods that focus only on generating the backbone structure, frame orientation allows us to implicitly account for side-chain positioning by encoding critical spatial relationships between residues. This orientation not only informs the side-chain arrangement but also reflects the interactions between residues, which are essential for maintaining the stability and function of the antibody. By leveraging frame orientation, we enhance the model’s ability to represent realistic protein structures, and after generating the backbone, we can apply classical tools like Rosetta to further optimize the side-chain conformations for increased accuracy and reliability.


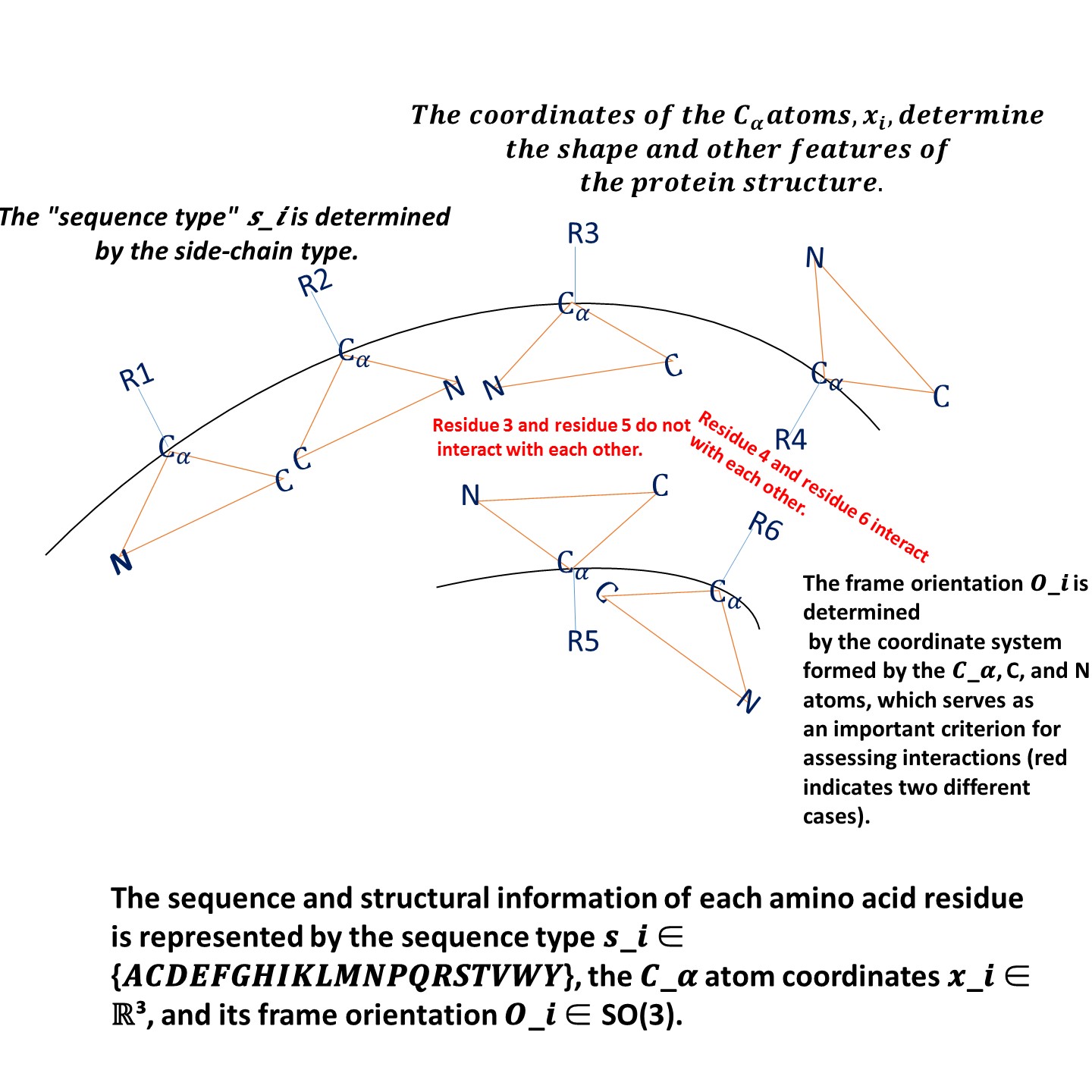
 **Supplementary Figure 4: frame orientation plays a crucial role in both encoding side-chain information and capturing inter-residue interactions**

**2.4 Future Directions**

In future work, we aim to publicly release the AlphaPanda algorithm and code to encourage collaboration and experimental validation. This collaborative approach will help verify the effectiveness of AlphaPanda, refine its parameters, and ultimately enhance its reliability for practical therapeutic antibody design. By involving a broader scientific community, we hope to bridge the gap between computational predictions and experimental validation, bringing us closer to real-world applications in biotherapeutics.
